# Biomineralization of Nickel Struvite Linked to Metal Resistance in *Streptomyces mirabilis*

**DOI:** 10.3390/molecules27103061

**Published:** 2022-05-10

**Authors:** Flávio Silva Costa, Falko Langenhorst, Erika Kothe

**Affiliations:** 1 Institute of Microbiology, Friedrich Schiller University Jena, Neugasse 25, 07743 Jena, Germany; flavio.silvacosta@uni-jena.de; 2 Institute of Geosciences, Friedrich Schiller University Jena, Carl-Zeiss-Promenade 10, 07745 Jena, Germany; falko.langenhorst@uni-jena.de

**Keywords:** biomineralization, struvite, nickel, streptomycetes, heavy metal resistance

## Abstract

Biomineral formation is a common trait and prominent for soil Actinobacteria, including the genus *Streptomyces*. We investigated the formation of nickel-containing biominerals in the presence of a heavy-metal-resistant *Streptomyces mirabilis* P16B-1. Biomineralization was found to occur both in solid and liquid media. Minerals were identified with Raman spectroscopy and TEM-EDX to be either Mg-containing struvite produced in media containing no nickel, or Ni-struvite where Ni replaces the Mg when nickel was present in sufficient concentrations in the media. The precipitation of Ni-struvite reduced the concentration of nickel available in the medium. Therefore, Ni-struvite precipitation is an efficient mechanism for tolerance to nickel. We discuss the contribution of a plasmid-encoded nickel efflux transporter in aiding biomineralization. In the elevated local concentrations of Ni surrounding the cells carrying this plasmid, more biominerals occurred supporting this point of view. The biominerals formed have been quantified, showing that the conditions of growth do influence mineralization. This control is also visible in differences observed to biosynthetically synthesized Ni-struvites, including the use of sterile-filtered culture supernatant. The use of the wildtype *S. mirabilis* P16B-1 and its plasmid-free derivative, as well as a metal-sensitive recipient, *S. lividans*, and the same transformed with the plasmid, allowed us to access genetic factors involved in this partial control of biomineral formation.

## 1. Introduction

Biomineralization in bacteria mostly occurs by induced biomineralization [1], with direct biomineralization referring to spatial and temporal, as well as macroscopic shape control [2,3], or forced biomineralization, which is not necessarily linked on a small scale spatially, but specifically requires biomolecules for heavy metals and radionuclide incorporation [4].

Molecular mechanisms in mineral formation are known to occur through the production of nucleation molecules, or by changing environmental conditions on a small scale [5,6,7,8,9]. Different molecules are involved as templates for the nucleation of biominerals [10], including proteins, lipids, or polysaccharides. These may be secreted and dispersed by diffusion, be parts of the cell wall, or be attached to the cell membrane [11,12,13]. In addition to bacterial cell wall components, extracellular polymeric substances (EPS) or S-layers, present, for example, on dormant spores, can act as a site for mineral nucleation and growth [3,14].

*Streptomyces* is a ubiquitous genus of filamentous soil bacteria, with *S. mirabilis* P16B-1 isolated from a former uranium mining site near Ronneburg, Germany, tolerating up to 130 mM of nickel when growing on solid minimal media [15]. Aside from rare earth elements and radioisotopes, nickel is, along with zinc, a major contaminant in this area. Nickel seems very interesting for studying specific biological mechanisms, since it is—in contrast to other heavy metals such as uranium—essential at low concentrations (by incorporation into the active center of enzymes like a specific superoxide dismutase) and toxic only at high concentrations. This dual role of nickel necessitates specific cellular mechanisms ensuring homeostasis. The isolate *S. mirabilis* P16B-1, with its exceedingly high resistance against specifically nickel in its environment, warrants to concentrate on the role of nickel in extracellular resistance mechanisms such as biomineralization. The contribution of plasmid-encoded functions to metal resistance has previously been shown [15,16]. The genome sequencing (GenBank acc. No. CP074102 for the chromosome and CP074103 for the large linear plasmid) of this extremely heavy-metal-resistant soil isolate revealed a giant, linear plasmid that was shown to harbor genes for metal transporters, including *nreB*, which is not found on the bacterial chromosome—only the plasmid contains a homologous sequence—and which codes for a nickel-specific efflux pump known to confer nickel resistance [17,18]. Here, we show that with the formation of green biominerals, nickel-containing struvite is potentially seen, which is enhanced when the plasmid is present in either *S. mirabilis* or *S. lividans*. This has been verified by producing a strain that does not harbor the plasmid *S. mirabilis* Δp [18]. Additionally, a knock-out of *nreB* on the plasmid was achieved that still produced crystals, but at lower incidence [18]. Hence, the plasmid contains information relevant for crystal production, in addition to the increased cell efflux through *nreB*.

Green Ni-struvite has previously been observed with another streptomycete isolated from the same area. On both liquid and solid media, mineral grains could be observed on mycelium or in close proximity to the growing colonies with *S. acidiscabies* E13 [19]. These crystals were identified to possess a struvite structure and to incorporate high concentrations of nickel, substituting for magnesium in the crystal structure. The Ni-struvite was insoluble in water or media and allowed for a reduction in soluble and hence bioavailable nickel concentrations. Thus, it may play an important role in the metal resistance of streptomycetes.

The mineral struvite is a hydrous magnesium ammonium phosphate (NH_4_)Mg(PO_4_) · 6 H_2_O, which precipitates from high ammonia and phosphate solutions. This results in the important process of precipitating struvite from phosphate-rich wastewater and re-using the phosphate for fertilization; in this context, it is advantageous that struvite can be biomineralized even at low supersaturation conditions [20]. These authors also described bio-struvite formation by an actinobacterium, *Mycobacterium* sp., and even found evidence for control over crystal growth with some of their strains. Lately, the involvement of bacterial siderophores has been shown to play an important role in the process [21]. This is also of interest with respect to metal-resistant streptomycetes, as the concomitant production of three siderophores has previously been shown for *S. acidiscabis* E13 as well as *S. mirabilis* P16-B1 [22,23]. As both strains have been seen to produce the green Ni-struvite, we investigated whether white struvite can also be precipitated in media devoid of nickel, and whether the occurrence of minerals is connected to the nickel transporter gene *nreB*. To that end, we addressed the question of whether plasmid contributed to biomineralization, and whether solely the nickel-containing mineral was formed by *S. mirabilis*, or alternatively, whether conditions could be described that promote the incorporation of a new metal ion replacing the naturally occurring magnesium of struvite. We show here that heavy-metal-resistant *S. mirabilis* P16B-1 induces the formation of struvite and, in the presence of nickel, incorporates nickel into the forming mineral, creating Ni-struvite. This process is discussed with respect to nickel resistance, and whether the concept of forced biomineralization within biologically induced mineral formation can be supported with the results obtained.

## 2. Results

### 2.1. Streptomycetes Are Able to Induce Crystallization

**Figure 1:**
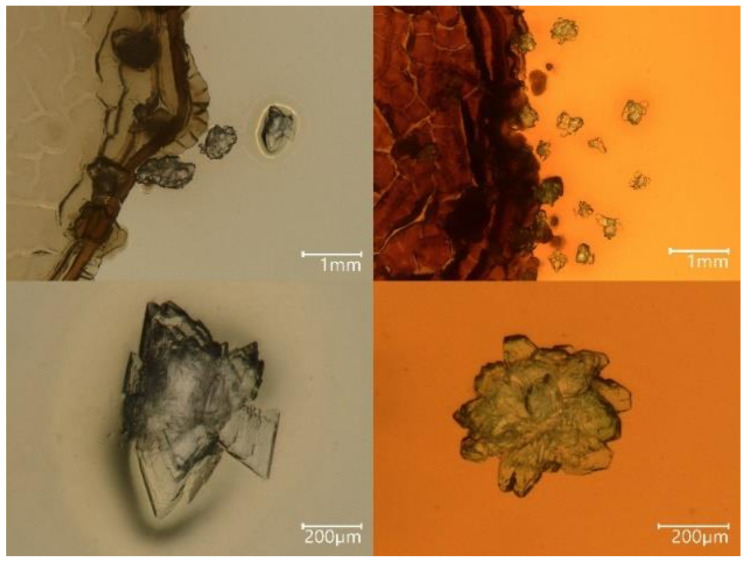
Crystals produced by *S. mirabilis* grown on solid media. Crystals in media without additional nickel (**left**) were compared to those produced if 10 mM NiSO_4_ had been added (**right**).

Although *S. mirabilis* P16B-1 is able to still show some growth at higher concentrations, we used media containing 20 mM of NiCl_2_ or NiSO_4_ here. At these concentrations, both salts and growth in liquid as well as growth in solid media allow for the maximum growth rates of the wildtype. Mineral formation was checked without and with added nickel salts. Using solid media, crystals were detected on or close to the biomass of the metal-resistant *S. mirabilis* (Figure 1). Without the addition of nickel, translucent, white crystals were observed, and upon increasing nickel availability, green mineral grains replaced the white ones. The green color in very small crystals was scored by observation in the binocular; green was scored independent of the deepness of color, as this was also dependent on crystal size.

To measure the yields of minerals, the crystals were removed and pooled from five biological replicates, and the weight was determined (Table 1). Without additional nickel, 4 mg of white crystals were obtained; both nickel salts increased the yield by at least 3-fold, in addition to the change in color. Since growth was impeded strongly in 20 mM NiCl_2_, higher nickel concentrations could not be applied, thus limiting the crystals’ yield.

Due to the previously discussed control of the large, linear plasmid of *S. mirabilis* P16-B1 on metal tolerance, we also checked whether the plasmid would participate in biomineral formation. Therefore, a cured *S. mirabilis* ΔpI was tested under similar conditions. Indeed, the cured strain led to a highly reduced biomineralization as well as reduced growth in media containing higher nickel concentrations (see Table 1). To investigate the molecular basis of this effect, we used a transformation of the model streptomycete *S. lividans* TK24 and compared the wildtype of this species to an exconjugant shown to contain the plasmid from *S. mirabilis* P16B-1, *S. lividans* pI. The plasmid in the receiver strain not only increased growth at elevated nickel concentrations but, in addition, increased the formation of minerals as observed in liquid media (Supplementary Figure S1).

No crystals were observed in any of the media without inoculation, or when inoculated with dead biomass or dead spores, while active growth increased mineral formation with increasing nickel availability. The crystals formed in aggregates and were green when produced in media containing more than 5 mM NiSO_4_ or NiCl_2_, while white crystals were obtained below that threshold, with sizes varying between 0.2 and 3 mm, independent of the strains (*p* = 0.197) or nickel salt (*p* = 0.06) used (Supplementary Figure S2).

### 2.2. Identification of Struvite Crystals

**Figure 2:**
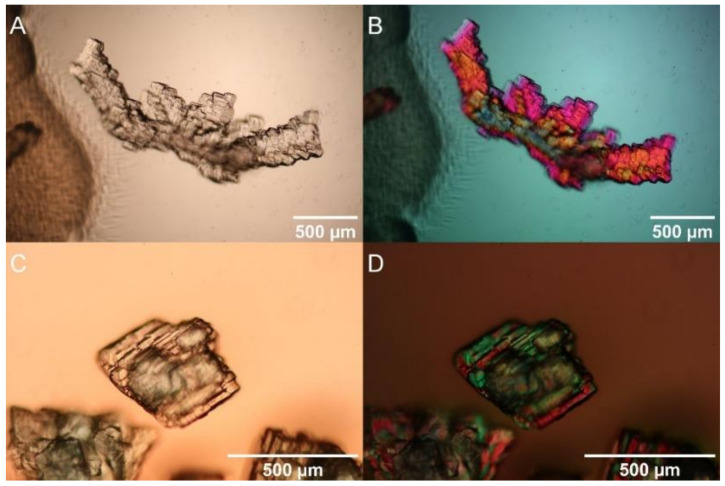
Polarized microscopy of crystals produced by *S. mirabilis*. Crystals produced by the wildtype in TSA (**A**,**B**) or TSA + 10 mM NiSO_4_ (C, D) were compared by light microscopy (**A**,**C**) and under polarized light (**B**,**D**).

The identification of crystals was based on polarizing microscopy, Raman spectroscopy, and energy-dispersive X-ray (EDX) spectroscopy on an analytical TEM. Polarizing microscopy revealed that the crystals possess clear crystallographic faces and colors varying from white transparent for Ni-free control experiments, to green for experiments with Ni-containing solutions. Under crossed nicols, the grains showed interference colors proving their birefringence and perfect crystallinity (Figure 2).

**Figure 3:**
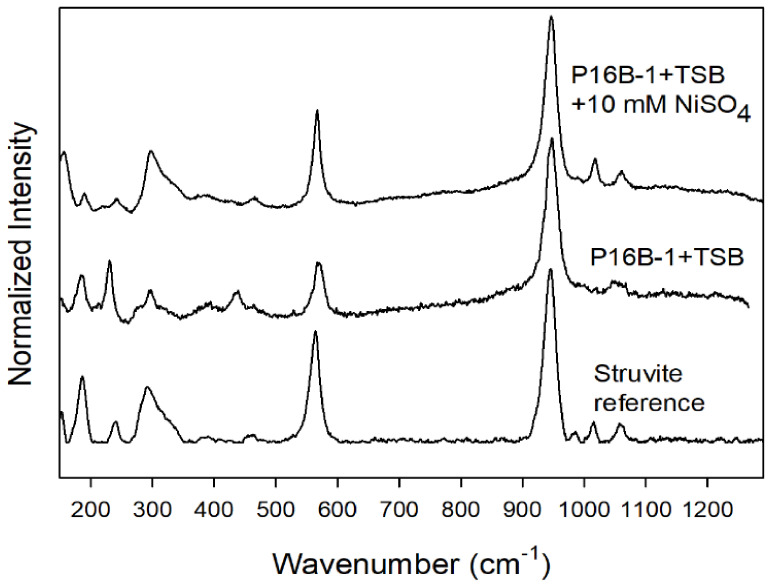
Identification of struvite by comparison of Raman spectra of crystals produced on TSB medium with or without nickel addition by *S. mirabilis* P16B-1, compared to struvite spectrum available in RRUFF database. For comparison, we have rescaled the spectra by normalizing the strongest Raman band at 946 cm^−1^ to an intensity value of 1.0.

Raman spectra obtained on the white transparent crystals produced without nickel perfectly resemble those of reference struvite (Figure 3). The main band at 946 cm^−1^ can be assigned to the stretching vibrations of the phosphate group in struvite. Raman spectroscopy performed on the green Ni-bearing crystals turned out to be more complicated, as the crystals decomposed during the measurements even at the very low (3 mW) power on the laser beam, resulting in dry ammonium-free phosphates. Reliable Raman spectra could, however, be obtained on green crystals. These spectra are again identical to the reference spectra of struvite, with the main stretching band at 946 cm^−1^.

**Figure 4:**
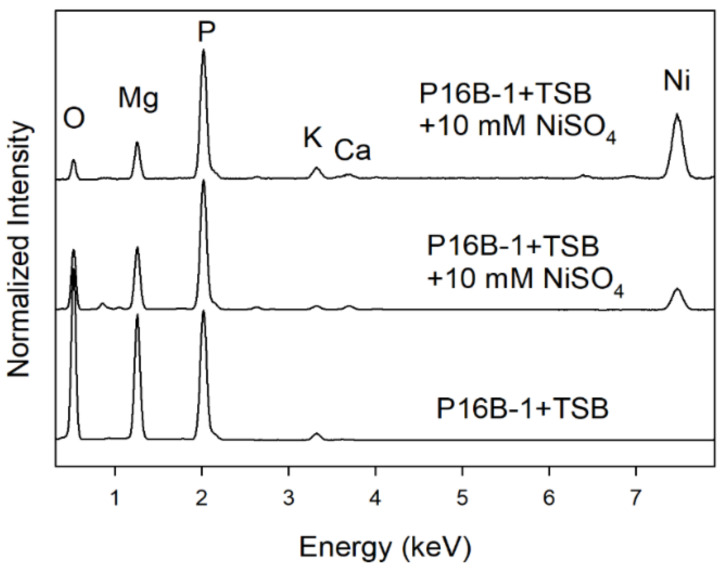
EDX spectra of *S. mirabilis* P16B-1 crystals produced with 10 mM NiSO_4_ (green) and without nickel addition (white) on TSB medium. For comparison, we have rescaled the spectra by normalizing the phosphorus K peak to an intensity value of 1.0.

In the course of the TEM investigations, we also noticed the instability of struvite crystals in that they immediately amorphized under high vacuum conditions. Thus, a structural analysis by selected area electron diffraction was not possible. Despite this, we were able to determine the chemical compositions of the biominerals by energy-dispersive X-ray spectroscopy. These analyses prove that the white crystals are Ni-free struvites, which mainly contain Mg on the B site of the general struvite formula A^1+^B^2+^[PO_4_] ∙ 6 H_2_O with A = NH_4_^+^ and B = Mg^2+^. A quantification of EDX analyses showed that trace amounts of K (on A site) and Ca (on B site) are present (Figure 4). EDX measurements also reveal variations in the Ni content of struvite crystals produced in experiments with Ni addition. In Figure 4, we illustrated this by showing two EDX spectra of Ni-containing crystals, in which the divalent Ni replaces Mg. Quantification of spectra shows a variation of compositions from (NH_4_^+^)(Mg_0.8_Ni_0.2_)[PO_4_] ∙ 6 H_2_O to (NH_4_^+^)(Mg_0.4_Ni_0.6_)[PO_4_] ∙ 6 H_2_O. Some trace amounts of K and Ca are present as well.

### 2.3. Biomineralization of Struvite and Ni-Struvite Is Promoted in Sterile Culture Supernatant

**Figure 5:**
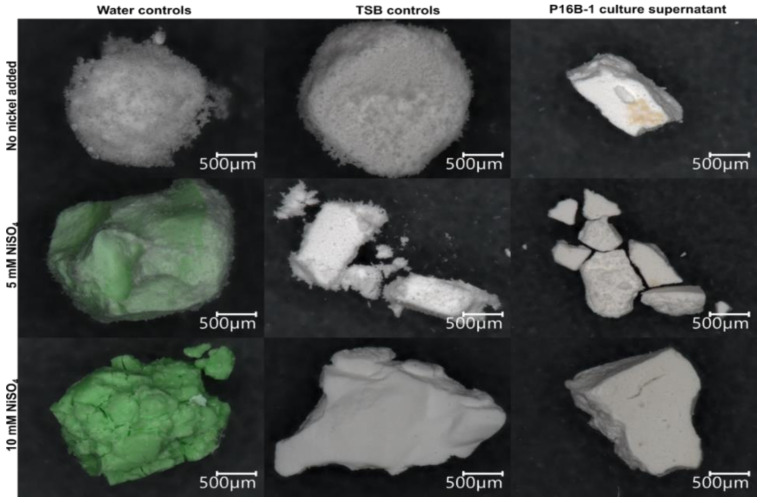
Biomimetically synthesized struvite crystals. Water and TSB medium controls were compared to sterile supernatant of *S. mirabilis* P16B-1 culture grown on TSB at different NiSO_4_ concentrations.

The formation of biominerals, both Mg- and Ni-struvite, was only possible with growing bacteria and not when dead biomass was added to the media. The TSB medium is undersaturated for struvite before bacterial growth; the calculated saturation index (SI) is −0.99. Biomimetic synthesis of struvite failed to mimic the habit (or characteristic external shape) of crystals precipitating natively. Here, the crystals formed in cell-free supernatant of media with or without nickel were similar to the ones precipitated in non-inoculated medium control (Figure 5; for overview see Supplementary Figure S3). None of the precipitates were green, except for different crystal shapes in pure nickel salt solution without culture medium or bacterium. Furthermore, in this case (where a green precipitate was obtained) the precipitation occurred when the pH reached ~5 instead of 9, which would usually be necessary for struvite synthesis.

With living *S. mirabilis* P16B-1 grown in liquid media, crystals were found attached to the bacterial mycelia (Supplementary Figure S4). Thus, a process depending on nucleation at the cell wall was one potential mechanism, albeit only if associated with some metabolic activity present during cellular growth. To obtain more information on the processes involved, sterile filtered cell-free supernatant was used. This led to the formation of crystals, both with incubation at room temperature, and after freezing and thawing of the samples, indicating a nucleation independent of the intact cell wall and associated with smaller, secreted molecules (see Supplementary Figure S4).

Crystals precipitated at room temperature were larger than the ones precipitated at freezing temperature (see Supplementary Figure S4). Again, media containing 5 or 10 mM NiSO_4_ led to the precipitation of green Ni-struvite, while media without additional nickel led to white Mg-struvite mineralization. This indicates that control over nickel incorporation, as well as the mineralization process, depends on secreted molecules present after bacterial growth in the culture supernatant.

### 2.4. Precipitation of Ni-Struvite Removes Nickel from the Water Phase

The formation of a nickel-containing mineral should reduce the bioavailable, water-soluble amount of nickel surrounding the cells and thus provide protection against excess nickel concentrations. We therefore measured whether the effect would indeed, even in high-nickel media, lead to nickel removal. An average of 5.41 mg/L Ni or 1.6% was removed when 5 mM NiSO_4_ (measured in the sample with 335 mg/L Ni) was added to the medium, while at 10 mM (or 590 mg/L) NiSO_4_, the difference was not measurable with statistical significance (Supplementary Figure S5).

Since with biomass present, the measurement cannot distinguish between uptake, adsorption, and biomineral formation, we repeated the experiment with the precipitation of crystals from cell-free culture supernatant. Here, a significant reduction in nickel ions was observed when 10 mM NiSO_4_ was present, with 24.5 mg/L (or 4.2%) Ni removal (Supplementary Figure S5). In sum, both processes of bacterial growth and subsequent mineral precipitation in cell-free supernatant could remove 8.98 and 27.73 mg/L Ni, corresponding to 2.6 and 4.6 % of the 5 and 10 mM NiSO_4_, respectively. While mineral amount varied between 30 and 60 mg biomineral retrieved from 200 mL, the values were in rough accordance, showing at least similar orders of magnitude between nickel removal and incorporation into minerals. The concentration of Ca, Mg and P concomitantly decreased, in agreement with Ni/Mg-struvite mineralization (Supplementary Figure S6).

## 3. Discussion

*S. mirabilis* P16B-1 uses a highly nickel-specific efflux pump, NreB, in order to survive under extremely high concentrations of up to 130 mM nickel as a resistance mechanism [16,18]. The protein shows high similarity to known nickel efflux pumps such as *Cupriavidus metallidurans* CH34 NreB (see Supplementary Figure S7). A pump-driven nickel efflux will lead to a higher local nickel concentration at and around the growing mycelium. Thus, in the presence of nickel, the expression of this gene is prone to create a nickel supersaturation in the direct vicinity of the cells, which might be essential in Ni-struvite precipitation. In this strain, the *nreB* gene is located on the plasmid, which was not essential for the production of struvite, nor Ni-struvite, considering that plasmid-free strains also produced biominerals of both colors (white and green). On the other hand, plasmid-harboring strains produced more and bigger crystals when nickel was present. The presence of *nreB* and increased the availability of nickel in the cells’ surroundings because the export of nickel from the cell can lead to a higher Ni-struvite supersaturation, which is the main parameter controlling the size of the struvite [24]. This increase in nickel availability also led to the precipitation of more minerals, since nickel treatment resulted in a greater mass of crystals produced than in treatments without nickel. However, other factors encoded on the plasmid obviously are necessary, as the plasmid-free derivative could form struvite as well as nickel struvite, even if at lower yields.

A second feature is that struvites usually precipitate at pH 9. However, in our experiments, Ni-struvite precipitated at pH ~5 instead of 9. Indeed, we could show that pH rises in liquid cultures, and this might add to the biomineralization capacity along with the locally increased concentration in nickel. As the best-documented biological importance of nickel is its incorporation into the active center of urease, the production of ammonia and consequent pH increase can be directly linked to that [25].

The precipitation of biominerals can also work as a mechanism to tolerate toxic concentrations of heavy metals, since the metal is immobilized by storage in crystals [4,26]. In our experiments, the metabolic activity of *S. mirabilis* P16B-1 was not enough to substantially reduce the concentration of nickel in the medium containing 10 mM of NiSO_4_, in contrast to the media containing 5 mM of NiSO_4_. Nickel immobilization by microbial-induced calcite precipitation was also less effective in a higher initial metal load [27]. However, these tests were conducted in liquid media under agitation; therefore, the solution was homogenized, and no microenvironmental effects could form. In solid media, on the other hand, the influence of mineralization on microenvironment concentrations of metals should be higher. For example, bacteria can create pH gradients at approximately 10 mm away from the colony, increasing the pH by releasing ammonia in the medium [28]. Both these processes, together, can lead to struvite precipitation [29].

However, our experiments show biomineralization acting as a heavy metal resistance mechanism in culture. On the other hand, soils are very heterogeneous, and the availability of nutrients and ions is lower than in culture conditions, in addition to competitors and other stressors. Therefore, it is not possible to determine yet if the biomineralization of struvite occurs in soil based on our experiments. However, in sub-natural conditions, i.e., soil extract media, *S. mirabilis* P16B-1 is also able to produce struvite [30].

The precipitation of minerals by *S. mirabilis* P16B-1 seems to be dependent on the interaction of this microorganism and its habitat, considering that the morphology and chemical composition of minerals produced by this strain varied according to the medium. Since the precipitation occurs in the vicinity of the colony and not directly at the cell wall, which could work as nucleating site for the mineralization [31,32], molecules produced and exported by the bacterium may nucleate the crystal formation or interact with the growing crystal altering its habit (hence the characteristic external shape of the crystals). Different molecules are described as modulating the habit of minerals precipitating extracellularly. In the case of struvite mineralization, poly-aspartic acid or low-molecular weight peptides are responsible for the biogenic morphology [13,33]. For gold minerals produced by a strain of *Delftia acidovorans*, a non-ribosomal peptide facilitates the precipitation of the minerals [12]. This might be linked to the observation that siderophores enhance struvite bioprecipitation [21]. A change in micromorphology is also possible if organic molecules modify the growing nanomineral surface. It is still to be determined which molecules are modulating the habit of struvite precipitated by *S. mirabilis* P16B-1. However, since different crystal morphologies were observed in the given growth conditions, different molecules may be playing this role.

The morphology of crystals precipitated in our experiments varied from microscopic single crystals of different shapes in liquid media to macroscopic aggregates in solid media. Several factors can influence the habit of a crystal—the availability of ions; contaminants; stage of growth; space; etc.—and they can give evidence of the mineralization process [10]. The different morphologies observed in our experiments may reflect variations in supersaturation (pH, ions concentration) [24] or different stages of growth of the crystal [33]. Furthermore, mineral habits are influenced by media composition [34,35] and the conditions where the mineral is formed.

Although the precipitation of struvite here occurs by induced mineralization, some level of control seems to be achieved when one considers that Ni-struvite is precipitating on Ni-containing media, and this process was not reproduced without the bacterium. Our biomimetic synthesis of struvite in the supernatant of *S. mirabilis* P16B-1 grown in media containing nickel failed to produce Ni-struvite. In the biomimetic synthesis experiment, Mg, NH_4_, and PO_4_ are added to the solution, and the pH is adjusted immediately afterwards, which leads to the precipitation of the crystals. On the other hand, the bacteria concomitantly increase the concentration of Ni, as well as the pH during growth. The different time of interaction between nickel and the growing mineral could interfere with the incorporation of nickel to the mineral. However, it is yet to be determined how the incorporation of nickel into struvite occurs in this case.

The use of struvite mineralization in wastewater treatment has already been implemented [13,20,21]. With the observed nickel struvite biomineralization, a process not only removing high phosphate and magnesium concentrations to avoid eutrophy in wastewater receiving streams and lakes, but also treatment for nickel removal in bioremediation processes becomes feasible [24,29]. Here, the minerals can be easily precipitated without the need for the cell removal that would be necessary if mere bioadsorption processes were considered [27]. Thus, and although higher percentages of metal can be bound to cell walls, this will be cost-effective, as no filtering but mere precipitation is necessary. We can show once more that a better understanding of the processes involved in metal homeostasis will not only lead to new findings in cell biology of bacteria, but also allows for applications such as bioremediation [26].

## 4. Materials and Methods

### 4.1. Strains and Media

*S. mirabilis* P16B-1 and a plasmid-cured descendent *S. mirabilis* Δp were compared to the metal-sensitive model streptomycete *S. lividans* TK24 and a transconjugant carrying the plasmid from *S. mirabilis* P16B-1, *S. lividans* TK24 pI (for more information, see Supplementary Figure S8). The bacteria were cultivated in tryptic soy broth (TSB; BD Diagnostic, Heidelberg, Germany) with or without the addition of 1.8 % agar. For metal tolerance and biomineralization assays, sterile filtered NiCl_2_ · 6 H_2_O or NiSO_4_ · 6 H_2_O solutions were added at concentrations of 2.5, 5, 10, or 20 mM.

### 4.2. Biomineralization Tests

To test for biomineralization on solid media, bacteria were inoculated with 10 µL droplets of spore suspension (10^9^, 10^7^, 10^5^, or 10^3^ CFU/mL), and plates were kept at 28 °C for 15 days. For control, non-inoculated medium and autoclaved, dead biomass were incubated. In liquid medium, biomineralization with or without agitation (100 rpm) were compared. The presence of crystals was checked by light microscopy (Keyence, Neu-Isenburg, Germany).

To identify mechanisms for biomineralization, cell-free supernatant (obtained by sterile filtering) was used either directly at room temperature for one week, or after being kept at −20 °C and thawing at room temperature. Crystals were collected by centrifugation (4000× *g*, 3 min), washed with water, and air-dried.

To compare for abiotic mineral formation, biomimetic mineral precipitation was performed. For that, 6.51 g/L MgCl_2_ · 6 H_2_O, 3.42 g/L NH_4_Cl, and 3.68 g/L NH_4_H_2_PO_4_ were added to the supernatant of *S. mirabilis* cultures. The pH was adjusted to 9.0 using NaOH to induce mineral formation, and crystals were collected by centrifugation as described above. Using similar biomimetic mineralization conditions, culture supernatant or fractions of the same were added to address which molecular principle is involved in control over struvite versus Ni-struvite biomineralization.

To test for a reduction in bioavailable metal concentrations through mineral formation, the concentration of nickel was measured in the supernatant before and after the precipitation of minerals. Using simultaneous radial ICP-OES/MS spectrometers (725ES with CCD-detector, Agilent, Waldbronn, Germany), nickel, phosphorus, magnesium, calcium, and iron contents were measured. Ammonia concentrations were measured with the Spectroquant Ammonium Test (Supelco, Merck, Darmstadt, Germany). Struvite saturation index was calculated using PHREEQC [34].

### 4.3. Mineral Characterization

Minerals were extracted from solid media using a spatula and washed with distilled water before air drying at room temperature for mineral characterization. Sizes of crystals were measured using a digital microscope VHX7000 (Keyence, Neu-Isenburg, Germany).

Mineral identification was based on Raman spectroscopy. Raman spectra were acquired using a 531.95 nm emission laser light attached to a WITec alpha300 M+ microRaman spectrometer (Ulm, Germany). To suppress sample damage by the laser beam, we reduced the laser power to low values of ≤5 mW. Measured spectra were compared to reference spectra from the RRUFF database [36] using the software CrystalSleuth [37].

Analytical transmission electron microscopy was performed using a 200 kV FEI Tecnai G2 FEG (Eindhoven, The Netherlands) equipped with an Oxford 80 mm^2^ energy-dispersive silicon drift X-ray detector, and a Gatan UltraScan 2 k CCD camera (Gatan, Pleasanton, CA, USA).

### 4.4. Statistical Analyses

Statistical significance was checked using ANOVA and Tukey HSD post hoc tests after checking for normality with the Shapiro–Wilk test. All statistical analyses were conducted in R, where plots were obtained with the R packages ggplot2 and ggpubr.

## Figures and Tables

**Table 1:** Mineral yield (mg) from solid media.

		*S. mirabilis* P16B-1		*S. lividans* TK24	
Salt Concentration (mM)		Wildtype	Δp ^1^	Wildtype	pI ^2^
Control	0	4.1	0	0	0
NiSO_4_	2.5	<0.1	<0.1	1	0
	5	0.7	1.1 ^3^	<0.1 ^3^	<0.1 ^3^
	10	<0.1 ^3^	no growth ^4^	14	12.2 ^3^
	20	14.5 ^3^	no growth ^4^	no growth ^4^	(4.5) ^3,5^
NiCl_2_	2.5	1.2	1.6	1.6	0
	5	5.9	0	1.1 ^3^	1.3 ^3^
	10	11.7 ^3^	no growth ^4^	0	42.4 ^3^
	20	(3.7) ^5^	no growth ^4^	no growth ^4^	(11.2) ^3,5^

^1^ A strain cured for the single plasmid pI was obtained through application of heat stress; ^2^ conjugative transfer of the plasmid pI from the donor *S. mirabilis* P16B-1 to the recipient *S. lividans* TK24 was achieved; ^3^ cells shaded green indicate the color of the crystals produced; ^4^ no growth indicates that the sensitive strains without the plasmid did not show any colony growth; ^5^ brackets indicate reduced colony growth; mineral weight was obtained from 5 pooled biological replicates that had been analyzed for growth and color.

## Data Availability

Not applicable.

## Supplementary Materials

The following supporting information can be downloaded at: https://www.mdpi.com/article/10.3390/molecules27103061/s1, Figure S1: Crystals formed; Figure S2: Size of crystals formed by *S. mirabilis* at different concentrations of NiSO_4_ or NiCl_2_; Figure S3: Struvite crystals synthesized on water, TSB medium, and supernatant of *S. mirabilis* P16B-1 culture; Figure S4: Crystals precipitated from liquid media and cell-free culture supernatant; Figure S5: Nickel concentration in the supernatant after growth of *S. mirabilis* P16B-1 with 5 or 10 mM NiSO_4_; Figure S6: Concentrations of Ca, Fe, Mg, and K in the supernatant of *S. mirabilis* P16B-1 before and after precipitation of minerals; Figure S7: Protein sequence alignment of NreB proteins; Figure S8: Strategy for obtaining *S. mirabilis* Δp and *S. lividans* pI.

## Sample Availability

Samples of the compounds are not available from the authors.
