# Supplementary material for: Biomineralization of Nickel Struvite Linked to Metal Resistance in Streptomyces mirabilis
Source: Molecules. 2022 May 10;27(10):3061. doi: 10.3390/molecules27103061 (PMC9145468; doi:10.3390/molecules27103061)
Supplement: Supplementary file 1 [file molecules-27-03061-s001.zip › molecules-1674420-supplementary.pdf]

molecules

Supplementary File

Biomineralization of Nickel Struvite Linked to Metal Resistance in *Streptomyces mirabilis*

Flávio Silva Costa [1], Falko Langenhorst [2] and Erika Kothe [1,*]

[1] Institute of Microbiology, Friedrich Schiller University Jena, Neugasse 25, 07743 Jena, Germany; flavio.silva-costa@uni-jena.de
[2] Institute of Geosciences, Friedrich Schiller University Jena, Carl-Zeiss-Promenade 10, 07745Jena, Germany; falko.langenhorst@uni-jena.de
* Correspondence: erika.kothe@uni-jena.de; Tel.: +49-3641-949291

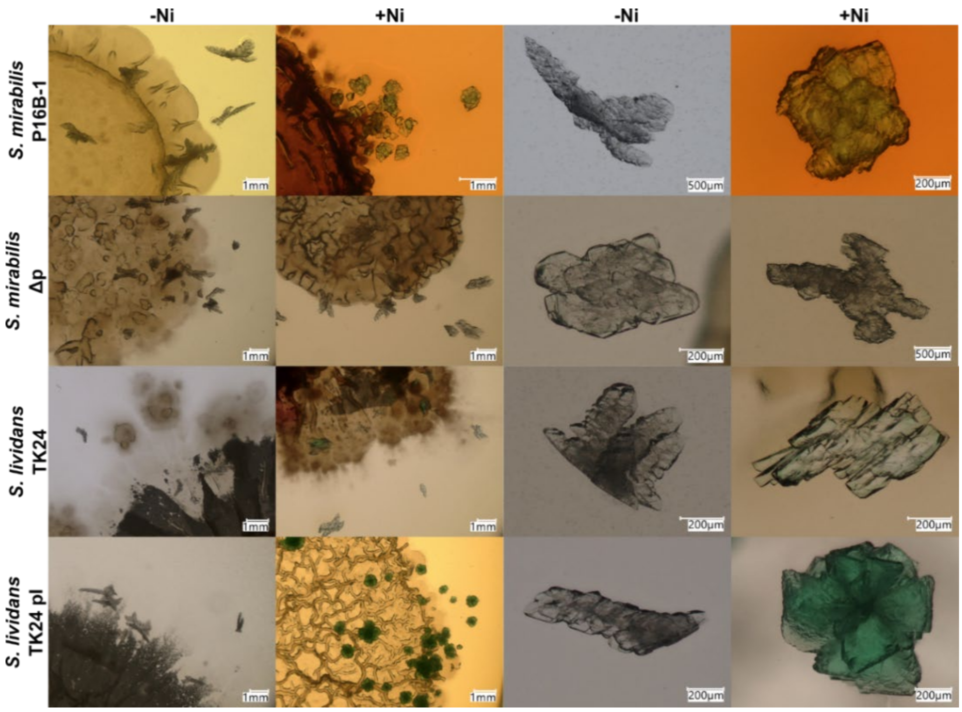

Figure S1. Crystals formed by the metal resistant *S. mirabilis* wildtype, the cured strain lacking plasmid pI, *S. mirabilis* DpI, and the metal sensitive strain *S. lividans* wildtype, as well as transfected with the plasmid pI from *S. mirabilis*, yielding *S. lividans* pI.

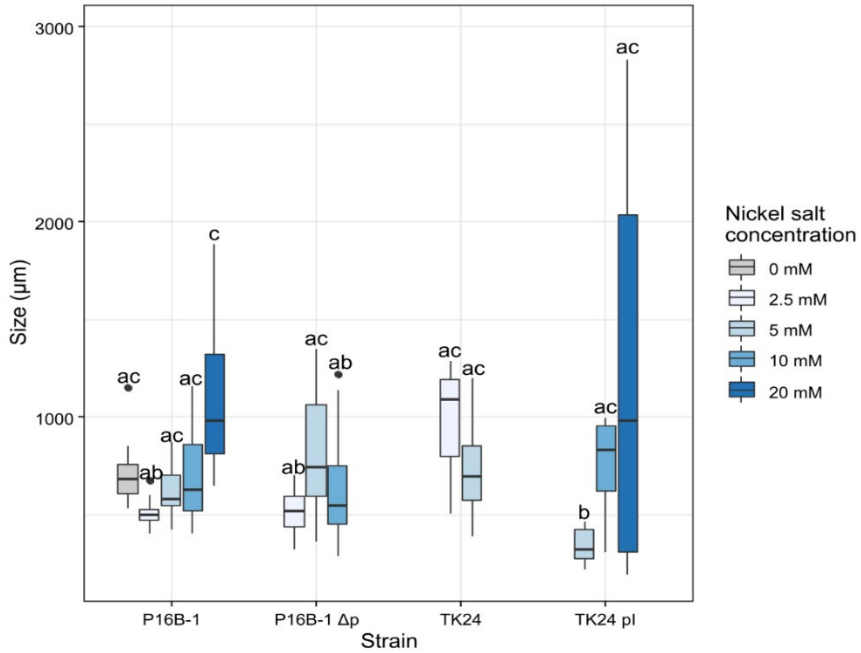

Figure S2. Size of 10 crystals obtained from the growth experiments (Tab. 1) formed by *S. mirabilis* P16B-1, the plasmid-free strain *S. mirabilis* DpI, the receiver strain *S. lividans* TK24 wildtype and the transformed *S. lividans* pI. Different concentrations of $NiSO_4$ or $NiCl_2$ are indicated by color. Differences between boxes with the same letter was not statistically significant (Two-Way ANOVA $p<0.001$, Tukey post-hoc test $p<0.05$, $n=10$).

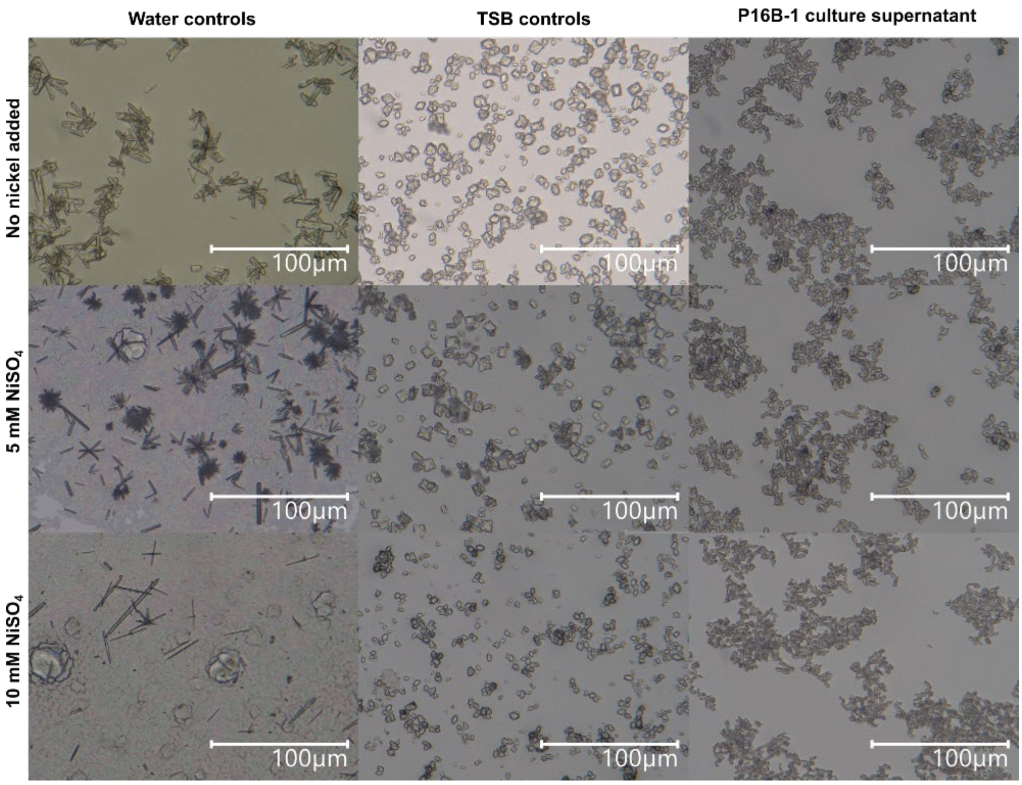

Figure S3. Struvite crystals synthesized on water, TSB medium, and supernatant of *S. mirabilis* P16B-1 culture.

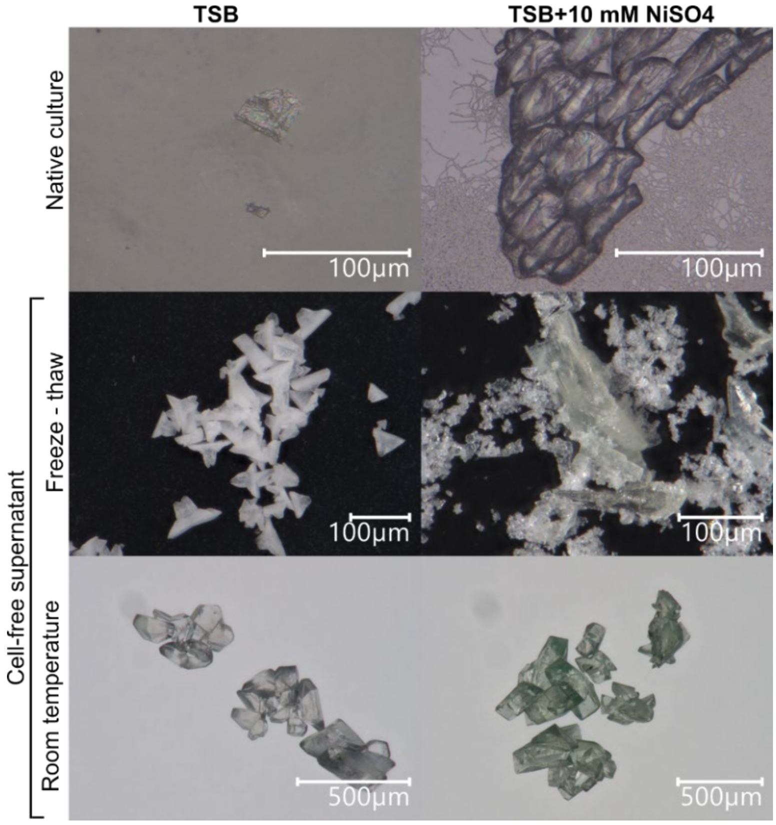

Figure S4. Crystals precipitated from liquid media and cell-free culture supernatant after one week of *S. mirabilis* P16B-1 growth. The effect of freeze-thaw cycles on crystal formation was evaluated.

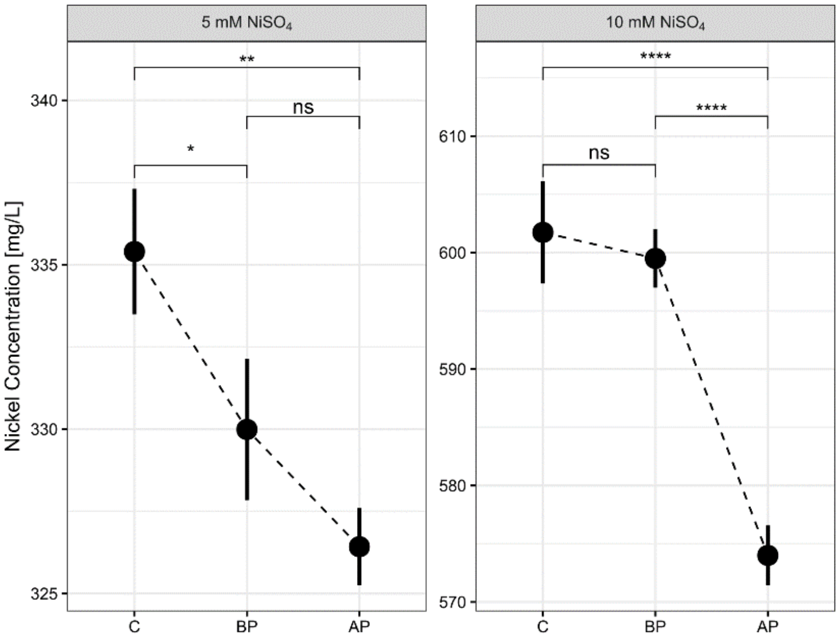

Figure S5. Nickel concentration in the supernatant after growth of *S. mirabilis* P16B-1 with 5 or 10 mM $NiSO_4$. For control, medium before cultivation was used (C), cell-free supernatant before crystal precipitation (BP) and after precipitation of crystals (AP) was measured. Statistical significance is indicated (ANOVA $p<0.05$; Tukey HSD test $p>0.05$ – not significant, ns; $p<0.05$ - *; $p<0.01$ - **; $p<0.001$ - ***; $p<0.0001$ - ****; $n = 4$).

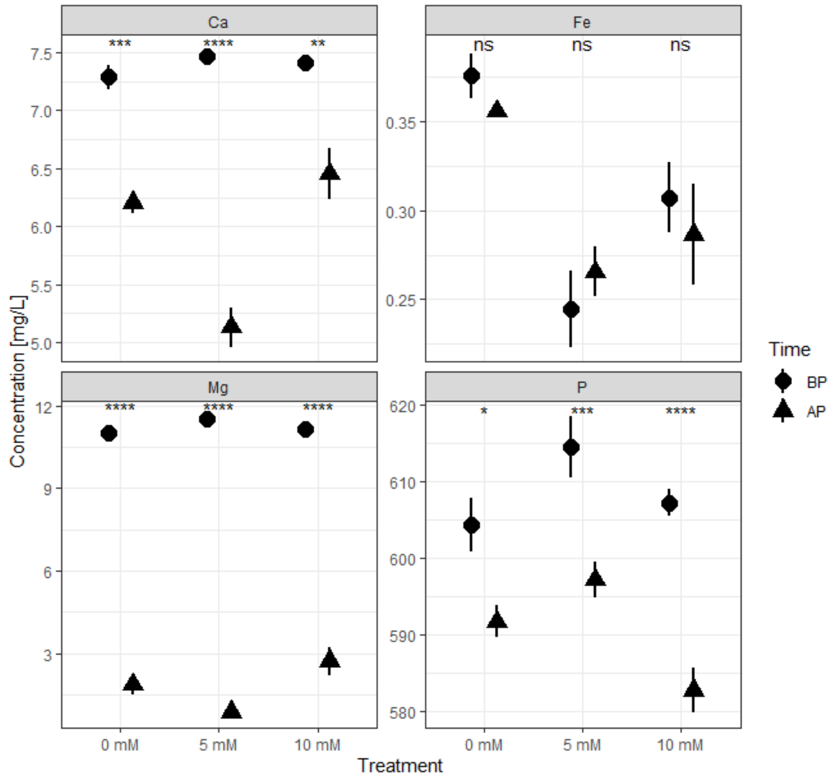


```
ies            Positives          Gaps
00(63%)  299/400(74%)  3/400(0%)

                  GADAGSVLGTALAIKMVAYVAI  61
                  GA+AGSVLGTALAIKMVAYV I
                  GANAGSVLGTALAIKMVAYVGI  60

                  QVYVLIFLLQAASAAFTPTFQA  121
                  Q+Y+LIFLLQ+ASAAFTPTFQA
                  QIYILIFLLQSASAAFTPTFQA  120

                  SLITYNWLFLGTVVGFLASAAL  181
                  S+I ++WLF GTVVGFL SAA
                  SVIGFHWLFGGTVVGFLVSAAF  180

                  ALLALNLAVAAASAMVTVNSVV  241
                   LLALNLA AAAS+MV VN+VV
                  GLLALNLAAAAASSMVIVNTVV  238

                  SDRVVMLSGALLLTVVFAGLGA  301
                   DR  ML GA+L++    A  G
                  PDRPAMLGGAILMSASLA-FGV  297

S. mirabilis      ITTAGSGGWRWGALLATWAAFGAACSMVLTPTGRLIRRAAPPEERTSAFAAQFSLSHSCW  361
                    T       W  LL TW A G   SM LTP+GRL++R+A    +R + FAAQFSLSH+CW
C. metallidurans  ALTGFGQVIEWPMLLLTWFATGIGYSMTLTPSGRLLKRSAASADRPAVFAAQFSLSHACW  357

S. mirabilis      LLTYPLAGWLGAAAGLQSAVLALGMIALFAALLAVRLWPS  401
                  L+ YPLAG +GA  G+++A + L  +++    L A R+WP+
C. metallidurans  LIAYPLAGQVGARYGMRAAFVVLACVSVVGFLAAWRVWPA  397
```

Figure S6. Concentrations of Ca, Fe, Mg, and P in the supernatant of *S. mirabilis* P16B-1 before (BP) and after (AP) the precipitation of minerals. Statistical significance is indicated (ANOVA $p<0.05$; Tukey HSD test: $p>0.05$ not significant, ns; $p<0.05$-0.011, *; $p<0.01$-0.011, **; $p<0.001$-0.00011, ***; $p<0.0001$, ****; n = 4).

Suppl. Fig. S7: Protein sequence alignment of NreB proteins. An alignemnt (BLAST P, https://blast.ncbi.nlm.nih.gov/Blast.cgi) was performed showing 63 % sequence identity between *S. mirabilis* P16B-1 NreB (SMIR_42025) and *C. metallidurans* NreB (acc. no. Rmet_6144).

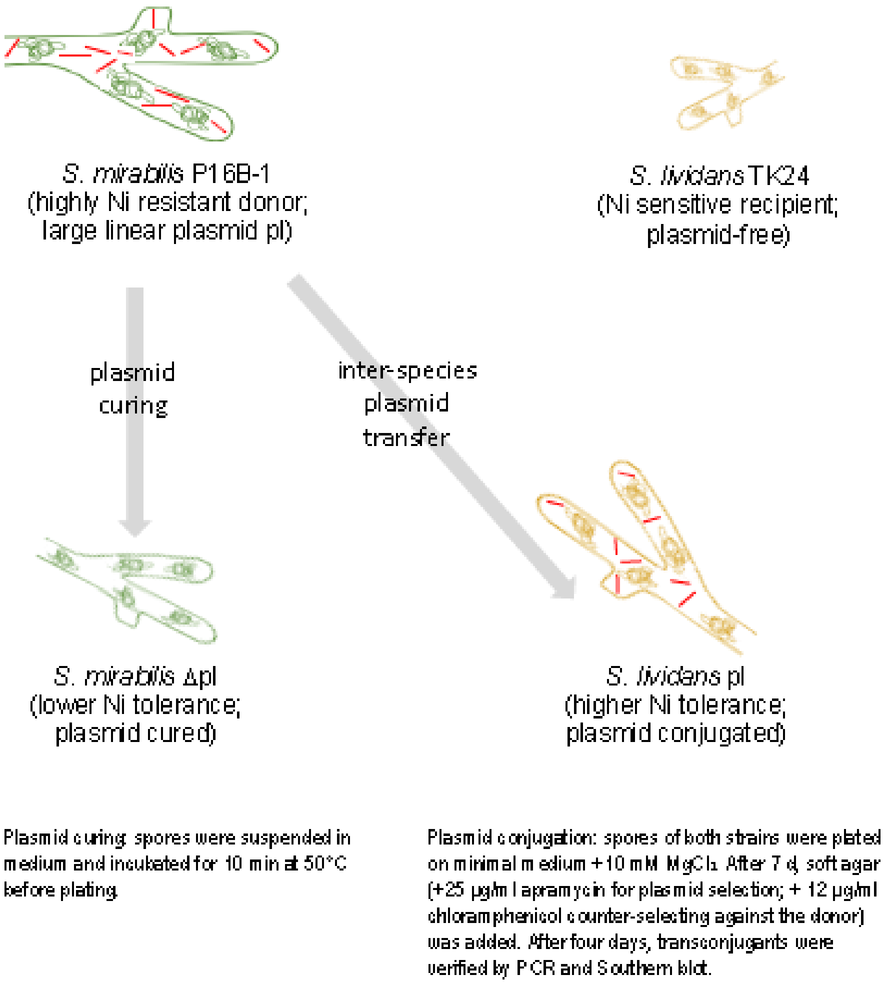

Figure S8. Strategy for obtaining *S. mirabilis* Dp and *S. lividans* pl. The representation shows the donor in green and the recipient in yellow; both filaments and the genome are indicated in the respective color. In addition, the and the large, liner plasmid pl is shown in red in the mycelia. The size of each mycelium indicates the tolerance against nickel in the medium. The experimental procedure is given below.
